# Supplementation of the Plant Conditioner ELICE Vakcina^®^ Product with β-Aminobutyric Acid and Salicylic Acid May Lead to *Trans*-Priming Signaling in Barley (*Hordeum vulgare*)

**DOI:** 10.3390/plants12122308

**Published:** 2023-06-14

**Authors:** Eszter Virág, Márta Kiniczky, Barbara Kutasy, Ágnes Nagy, József Péter Pallos, Levente Laczkó, Csongor Freytag, Géza Hegedűs

**Affiliations:** 1 Research Institute for Medicinal Plants and Herbs Ltd., Lupaszigeti Str 4, 2011 Budakalász, Hungary; kiniczky.m@gynki.hu (M.K.); aginagy.nagy@gmail.com (Á.N.); pallos.jp@gynki.hu (J.P.P.); hegedus.geza@zek.uni-pannon.hu (G.H.); 2 EduCoMat Ltd., Iskola Str 12A, 8360 Keszthely, Hungary; 3 Institute of Metagenomics, University of Debrecen, Egyetem Square 1, 4032 Debrecen, Hungary; laczko.levente@med.unideb.hu (L.L.);; 4 Department of Plant Physiology and Plant Ecology, Institute of Agronomy, Hungarian University of Agriculture and Life Sciences, Georgikon Campus, Festetics Str 7, 8360 Keszthely, Hungary; kutasy.barbara.julia@uni-mate.hu; 5 ELKH-DE Conservation Biology Research Group, Egyetem Square, 4032 Debrecen, Hungary; 6 Department of Information Technology and Its Applications, Faculty of Information Technology, University of Pannonia, Gasparich Márk Str 18/A, 8900 Zalaegerszeg, Hungary

**Keywords:** beta-aminobutyric acid, BABA, salicylic acid, *Hordeum vulgare*, biostimulant, RNA-seq, transcriptome, gene expression

## Abstract

Plant immunological memory, priming, is a defense mechanism that can be triggered by external stimuli, leading to the activation of biochemical pathways and preparing plants for disease resistance. Plant conditioners improve yield and crop quality through nutrient efficiency and abiotic stress tolerance, which is enhanced by the addition of resistance- and priming-induced compounds. Based on this hypothesis, this study aimed to investigate plant responses to priming actives of different natures, including salicylic acid and beta-aminobutyric acid, in combination with the plant conditioning agent ELICE Vakcina^®^. Phytotron experiments and RNA-Seq analyses of differentially expressed genes using the combinations of these three investigated compounds were performed in a barley culture to investigate possible synergistic relationships in the genetic regulatory network. The results indicated a strong regulation of defense responses, which was enhanced by supplemental treatments; however, both synergistic and antagonistic effects were enhanced with one or two components, depending on the supplementation. The overexpressed transcripts were functionally annotated to assess their involvement in jasmonic acid and salicylic acid signaling; however, their determinant genes were highly dependent on the supplemental treatments. Although the effects overlapped, the potential effects of *trans*-priming the two supplements tested could be largely separated.

## 1. Introduction

Crop production depends critically on the ability-adaptive response of plants to stressful conditions. Sustainable crop production focuses on new agronomic strategies to stimulate and strengthen plant response. Biostimulants can help plants rapidly cope with biotic and abiotic stressors and achieve a positive physiological state [1]. The research results on priming-active materials involving chemical or natural inducers in agricultural practice show good feasibility [2,3]. The effect of these agents is that they do not target pathogens such as pesticides or directly induce an immune response that is overcome by pathogenic microbes; however, they potentiate the long-term defense mechanisms of plants. When the stimulus and the stress are of the same type, we speak of “*cis*-priming or stress tolerance”, but when the priming and the triggering stimulus are different, we can speak of “*trans*-priming or cross-tolerance” [4,5,6]. To reduce the effect of osmotic stress, cowpea seeds were treated with polyethylene glycol (PEG) and β-aminobutyric acid (BABA) as priming elicitors. The use of PEG as *cis*-priming and BABA as *trans*-priming agents showed different physiochemical responses to PEG stress; BABA-primed seeds showed better regulation of osmotic stress [7].

Some natural and synthetic compounds, such as the non-protein BABA or the phytohormone salicylic acid (SA), have shown good priming-inducing activity under laboratory and field conditions [3,8]. In the last 20 years of studying these compounds, the different mechanisms of the plant defense system have also been increasingly brought into the light, paving the way for new plant protection strategies [9]. These different strategies depend on the required control of different attackers, such as insect herbivores and biotrophic and necrotrophic pathogens, and these are regulated by different signaling pathways controlled by phytohormones such as jasmonic acid (JA), SA, abscisic acid (ABA) or ethylene (ET) [10]. The increased production of these plant hormones can favorably influence the development of systemic resistance triggered by their exogenous addition [11] as a natural priming agent. Among the synthetic chemical priming agents, the non-proteinogenic amino acid BABA has been extensively studied due to its broad spectrum of activity [12]. Priming by SA and BABA can lead to resistance to biotic and abiotic stresses, with signaling pathways that can offset the physiological consequences of stress [13]. Several biostimulants used in agriculture as plant conditioning agents rely on the hormonal composition of the plant to provide stimulation through an exogenous influence [14].

Recently, the possible use of BABA as a plant biostimulant in monocotyledonous barley (*Hordeum vulgare*) crops was reported. Hegedűs et al. (2022) compared the stimulatory effects of this agent between monocotyledonous and dicotyledonous plants using in silico transcriptome profiling and pathway analysis. The results suggest an enhanced bacterial response, but a more specific stimulation of pathogen defense pathways was detected in *Arabidopsis thaliana* than in barley [15]. The synergistic interaction of BABA with fungicides was described by Cohen et al. (2002) [16]; therefore, its additional use in combination with plant conditioners seems promising in the control of plant diseases.

ELICE Vakcina^®^ (EL; alternative name, Elice16Indures) is a commercially available plant-extract-based plant strengthener distributed in the European Union and developed by the Research Institute for Medicinal Plants and Herbs Ltd. Budakalász, Hungary. The effect of EL on yield enhancement and plant vigor was studied and demonstrated in field crops of pea (*Pisum sativum*), oilseed rape (*Brassica napus*), soybean (*Glycine max*), and winter barley [17,18,19,20]. The yield increase triggered by EL was demonstrated by the instrumental measurement of hectoliter weight [20]. However, the higher resistance resulting from the priming effect could not be measured phenotypically in field crops. Transcriptomic studies showed enhanced hormonal signaling pathways, indicative of the priming state of plants. These suggestive priming effects have been described as triggers of systemic acquired resistance (SAR) and induced systemic resistance (ISR) in barley [20]. Hegedűs et al. (2022) found that these mechanisms underlie the induction of different hormones involving JA/ET-response- and SA-response/pathogenesis-related (PR) genes in response to low and high treatment doses, respectively. In addition, low- and high-dose treatments have been associated with hypothesized priming mechanisms, suggesting differences between the direct induction of hormone pathway genes and elicitor genes. Decsi et al. (2023) reported the genome-wide transcriptional profile of soybean cultures treated with EL. These data showed the inducibility of some immune response genes involved in the biosynthesis of JA, SA, isoflavonoids, phytoalexins, mitogen-activated protein kinase (MAPK) cascade, cellular detoxification, and oxidative stress response [19].

We hypothesize that the supplementation of EL with a small amount of BABA and SA could enhance these induced resistance mechanisms. To test this hypothesis, we grew barley (variety ‘SU Ellen’) used in field trials in a phytotron. In addition, we focused on the synergistic effect of the studied compounds BABA, SA, and EL. Differential gene expression analysis and functional annotation analysis for gene set enrichment showed that the effect of SA and BABA on EL changed, which probably led to *trans*-priming.

## 2. Results

### 2.1. Treatment of Barley Seedlings by Priming Active Agents

The experimental design was planned to investigate the complementary components of EL and their synergistic and antagonistic effects. Combinations of SA, BABA, and EL were investigated by collecting samples at two time points. Collected leaf samples were used to generate Illumina Gex libraries for RNA-Seq using the NextSeq550 sequencing platform. Gene expression differences were determined using pairwise DEGs and pathway analyses.

### 2.2. De Novo Assembly, Mapping, and Functional Annotation of Illumina RNA-seq Reads

**Figure 1:**
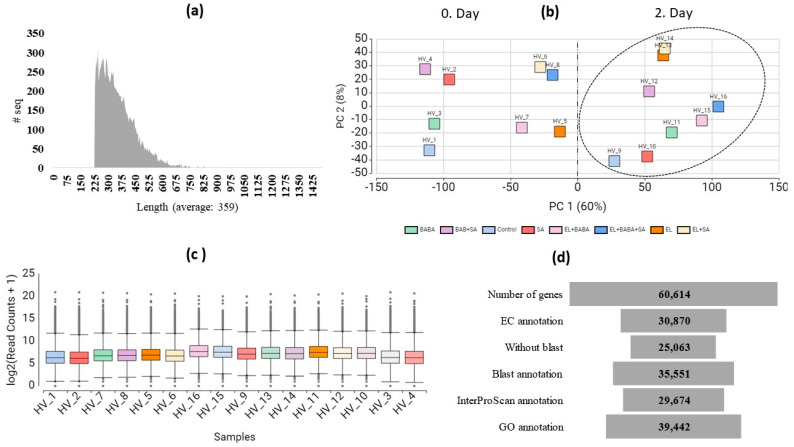
Distribution of reads, transcript abundances, and functional annotation of superTranscripts. The number of transcripts as a function of contig length (**a**), a scatter plot of PC1 and PC2 (PCA plot of read counts aligned to superTranscripts) explaining 60% and 8% of the variation, respectively, and separation of samples by treatments (**b**), distribution of read counts across samples (**c**), number of annotated sequences in the NCBI nr database (**d**). Because the treatments showed a significant difference on Day 2 compared to Day 0, see highlighted in an ellipse (HV _9- HV _16) on (**b**), we decided to include these data in the further analysis compared with the absolute control (HV_1).

The reference transcript dataset contained 73,301 nucleotide contigs (transcripts) with an average length of 359 bps and minimum and maximum lengths of 230 and 1475 bps, respectively (Figure 1a).

All reads of the 16 samples were mapped against the reference transcript dataset. Statistics of the mapped reads were performed and compared. Reads obtained under the same conditions were pooled as one sample. The assembly at the gene-level contained 60,614 superTranscripts, which were further analyzed.

A total of 58.65% of the reference transcripts could be functionally annotated by searching the NCBI nr database (Figure 1d). The entire annotated dataset showed an approximate functional distribution of gene ontology categories (GO). The annotation results were used to compare expression in single samples and single gene analyses. The combined de novo transcriptome of 16 samples, the number of transcripts read, and the annotation data were deposited in Mendeley Data (https://data.mendeley.com/datasets/68zy55gt62/1, accessed on 8 June 2023). Transcript abundances were performed at the gene level (referred to as superTranscripts), and principal component analysis (PCA) of normalized read counts was visualized and used to determine differences between treatment groups. Within the first two principal components, which accounted for approximately 60% (PC1) and 8% (PC2) of the observed variation (Figure 1b), groups were clustered by treatment. After segregation by treatment (Figure 1c), significant differences in counts were observed between treated groups on study days. Therefore, we chose to analyze samples from 1 (HV_1) vs. 10–16 (HV_1- HV_16), which represent the absolute control on Day 1 as opposed to all treatments on Day 2. The sample selection reflects the effect of EL-complemented well treatments compared to independent treatments with the priming agents used.

### 2.3. Pairwise DEGs and Fisher’s Exact Test

**Figure 2:**
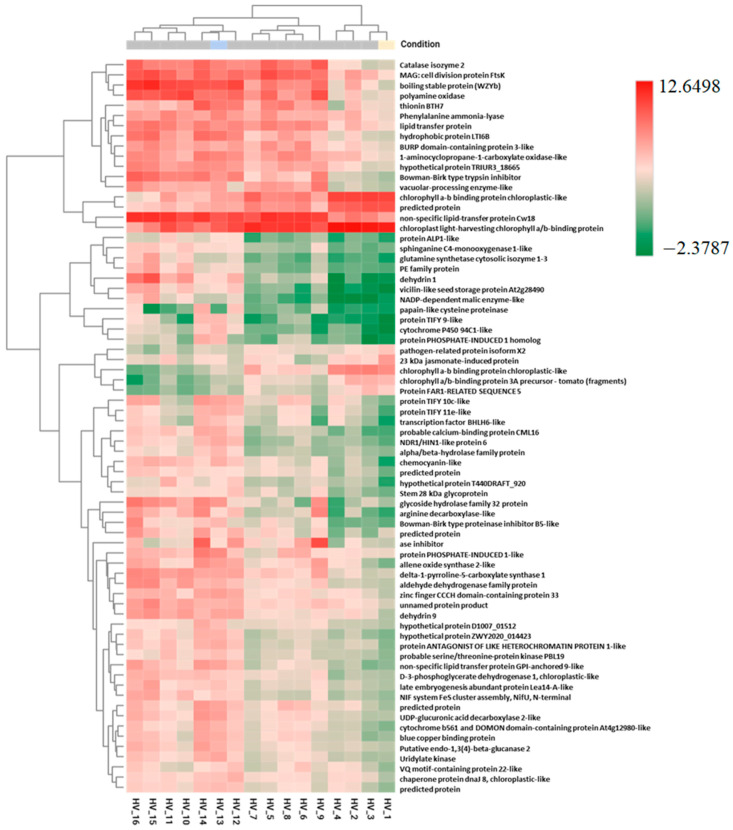
Two-dimensional heat map of the most significant DEGs of selected groups of 1 vs. 10–16. First, the top 50 DEGs of each sample pair were determined, yielding a total of 72 DEGs. Gene expression of these transcript IDs was visualized for all treatments. Test and reference genes were: the control (1) and the treatment with EL (13). Since all DEGs were considered, this heatmap effectively shows the effect of the additional treatments, either up- or down-regulated compared to EL. The dendrograms on the left and top were created by a hierarchical clustering method using the Euclidean distance calculated between the sequences (**left**) and samples (**top**) as input. When drawing the heatmap with the raw CPM counts, the log2 values were calculated, and the Z-score transformation was applied.

Pairwise analysis of differentially expressed genes (DEGs) was performed on samples from all treatments. All treated samples collected on Day 2 were compared with Day 1 control samples. The top 50 annotated DEGs were visualized in heatmaps, which indicated similar transcripts in 81% (see Figure S1). The IDs of all transcripts represented in these heatmaps were collected, analyzed and visualized again (1 vs. 13), resulting in 72 DEGs in all samples analyzed (Figure 2).

The genes downregulated by the treatments are mainly related to photosynthesis and light output. These are chlorophyll a/b-binding proteins and FAR1-related sequences. We identified 22 up-regulated sequences related to abiotic or biotic stress responses, including endo-1,3(4)-beta-glucanase 2 (PR2), Bowmann–Birk-type proteinase inhibitor (BBIs, PR6) genes, phenylalanine ammonialyase (PAL), and alkene oxide synthase (AOS), which are key enzymes of the biosynthetic pathways SA and JA (Table 1). The functional summary of the genes contributing to the stress responses of the top50 DEGs is summarized in Table S1. Because of the high expression of PAL, AOS, and JAZ proteins, we decided to analyze the signaling pathways JA and SA more intensively.

The TIFY transcription factor (TF) family proteins TIFY9, TIFY10c, and TIFY11e, which belong to the phylogenetic cluster of the jasmonate TIFY domain, were also among the top 50 DEGs, indicating a strong link to induced stress resistance. The overexpression of TIFY9, 10c, and 11e with the co-expression of AOS and PR6 were found only in EL-treated samples (13). EL samples treated with SA (14) and EL + BABA (15) had TIFY10c and co-expression of AOS, catalase2 (CAT2), and PR3. Strong downregulation of TIFY9, 10c, and 11e was detected in Samples 10 and 11 after the single treatments with SA and BABA with the concomitant suppression of AOS, suggesting that these materials do not affect the regulation of the JA pathway by TIFY-TF. An overexpression of PAL was found after all treatments (not only in SA-containing combinations), suggesting that the PAL pathway can also be induced by EL, SA, and BABA.

**Figure 3:**
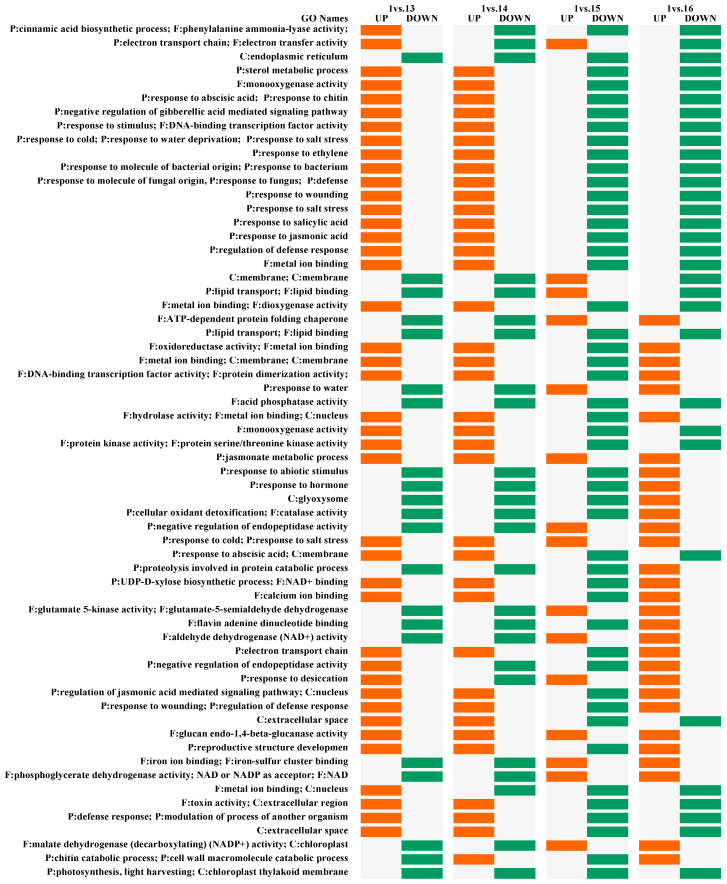
GO name distribution of over- and under-represented transcripts’ pairwise analysis of samples treated with EL and additional treatments (13–16) on Day 2 compared to absolute control (1) on Day 1. Shown based on FatiGO results using in-house software.

Fisher’s exact test was used to determine whether GO terms were over- or underrepresented in the genes in Sample 1 (reference) and the reference group of 16 (test). The up- and down-regulated genes were determined in comparison to the following reference: if the proportion of genes annotated with a particular GO term was significantly higher in the test group than the proportion in the reference group, this GO term was declared as being overrepresented (UP), and if otherwise, as being underrepresented (down) (Figure 3). We performed a statistical assessment of annotation differences between 2 groups of transcripts using FatiGO. More than half of the GO terms showed that the treatments stimulated the regulation of photosynthetic processes and defense responses. However, the regulation changed after the additional treatments, and antagonistic effects were observed, manifesting in the combined treatment of EL + BABA + SA. A positive regulation of response to monooxygenases, ABA, gibberellic acid (GA), ET, SA, JA hormones and also genes involved in response to fungi, bacteria, and wounding were overexpressed after treatment with EL and EL + SA.

Abiotic stress responses were also significant in these samples, including cold, water, salt, and desiccation stress. In contrast, most of the transcripts annotated with similar GO names were downregulated in the EL + BABA and EL + SA + BABA samples. We found that the significant cell wall reinforcement processes as part of the defense mechanism were also stimulated by EL + SA and AL + BABA + SA. Because these responses were underrepresented in the other two treatments, we hypothesize that the SA treatment may affect cell wall processes. All treatments examined restricted processes related to the chloroplast, thylakoid membrane, and light output.

### 2.4. Analysis of the Genes of the JA- and SA-Pathway

**Figure 4:**
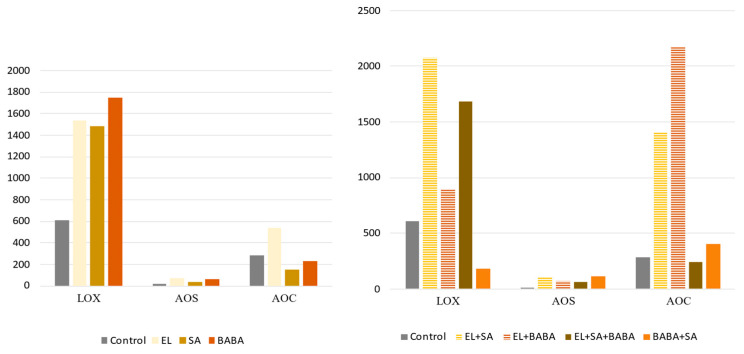
RPM values of the JA metabolism genes in response to the treatments studied on Day 2. The addition of the EL treatment had a synergistic effect on LOX, AOS, and AOC when SA and BABA were applied separately. The combined application of these priming agents worsened the effect of EL.

The top 50 DEGs and the GO terms indicated strong induction of SA and JA metabolic genes. Therefore, we selected the key enzymes of these metabolic pathways and identified the RPM values of lipoxygenases (LOX), AOS, and allene oxide cyclase (AOC) of the JA pathway (Figure 4).

**Figure 5:**
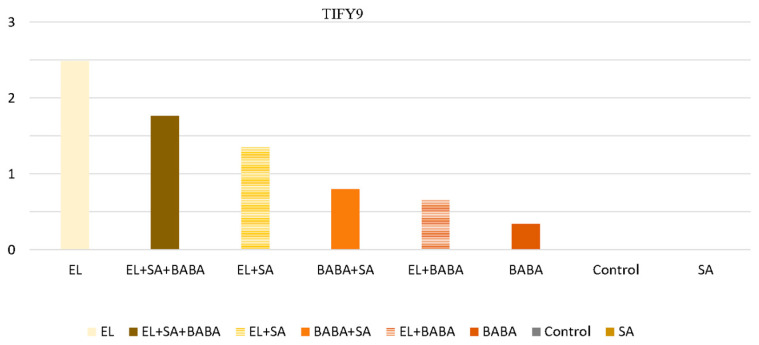
RPM values of TIFY9 genes induced by all treatments examined in Day 2. The data show that the inducing effect of EL on TIFY9 is reduced by additional treatments that does not affect TIFY9 transcriptional activity, either alone or in combination.

**Figure 6:**
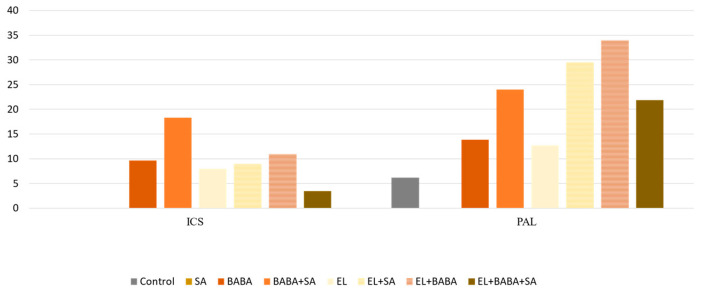
RPM values of ICS and PAL of SA pathway genes after the treatments studied on Day 2. Supplementation of EL with BABA and SA successfully increased the transcription of PAL and strengthened the PAL branch of SA biosynthesis.

In this study, all treatments were compared on the second day with the absolute control sampled on the first day. TIFY9 was also selected and found to play an important regulatory role in the biosynthesis process of JA (Figure 5), and was found to be triggered by EL. The key enzyme genes, isochorysmat synthase (ICS) and PAL, of the two branches of the SA pathway were also selected, and their RPM value was determined (Figure 6).

The results of the pairwise DEG and RPM analyses were compared, and the concordant changes observed in both analyses were summarized for the JA and SA pathway genes (Table 2).

## 3. Discussion

Research on biostimulants for plants is the focus of many agricultural fields. This involves microbial and non-microbial compounds that can be applied to seeds, soil or foliage to increase vigor, growth, and yield. Biostimulants stimulate plant nutritional processes, improve stress tolerance, nutrient use efficiency, and plant quality. These effects can mitigate various stressors, such as cold, drought, salt, or disease, which can cause plants to expend more energy on respiratory processes, thereby impairing photosynthesis. EL is a commercial plant conditioner with biostimulant activity. The advantage of this agent is that it contains plant CO_2_ extracts and can, therefore, be used in organic farming.

The stress-reducing effect of the product EL has been demonstrated in barley, canola, soybean, and pea [17,18,19,20]. Previous research on this compound suggests a priming-active property that stimulates ISR (via the JA/ET pathway) and SAR processes (via the SA and PR genes) in barley, which is due to its high phytohormone content [20]. The use of EL has been shown to activate plant defense signals, leading to massive transcriptional reprogramming in soybean [19]. Soybean treatment showed strong overexpression of genes for PR proteins, phytoalexins, hormonal signaling pathways, and various defense-related mechanisms, such as oxidative stress. To achieve an even better priming effect, we investigated two potent stimulatory compounds, SA and BABA, as potential complementary elements of the product EL. In this study, we summarize the synergistic and antagonistic effects of the combined treatment of EL with SA and BABA and investigate the gene expression profiles.

Pairwise DEG analysis revealed strong abiotic and biotic stress responses.

Analysis of the top 50 DEGs revealed that the expression pattern indicated strong regulation of various stress responses. Plant metabolism changed after the treatments, as evidenced by a reduction in photosynthetic processes associated with the down-regulation of the proteins’ chlorophyll a/b binding (LHCB) and the Far-red impaired response1 (FAR1). LHCB from the LHC family plays a role in energy-dependent quenching, which increases the thermal dissipation of excess absorbed light energy in the photosystem [26]. FAR1 is required for chlorophyll biosynthesis [27] and may be related to the expression of defense-responsive genes [28]. FAR1 binds to the ABA-Insensitive 5 (ABI5) promoter and activates its transcription, mediating ABA signal transduction and abiotic stress responses [29]. This correlation supports the concept that the priming-active compounds studied affect photosynthesis but do not activate ABA-related defense mechanisms. A significant upregulation of genes associated with biotic stress, such as papain-like cysteine protease (PLCP), thionin BTH7, and CAT2, was found. PLCPs are required for complete plant resistance to various pathogens [30], and are targeted by secreted pathogen effectors to suppress immune responses [31]. PLCPs are subject to a co-evolutionary arms race between host and pathogen [32], and can trigger a variety of defense responses, including plant cell death [33]. Thionins are plant-specific antimicrobial peptides that have been isolated from numerous plant species [34]. Their co-expression with CAT2 was observed in our studies and serves to protect cells from the toxic effects of hydrogen peroxide [35]. They are thought to be involved in the response to biotic stimuli [36] and plant immunity [37] through the biosynthesis of JA by facilitating the direct interaction of the JA biosynthetic enzymes acyl-CoA oxidase2 (ACX2) and ACX3. Yuan et al. (2017) substantiated their role in the biosynthesis of JA. They found that SA suppresses CAT2, leading to the inhibition of the accumulation of JA [38].

The enhancement of abiotic stress was manifested by the overexpressed aldehyde dehydrogenase (ALDH) family, bHLH-TF family (bHLH6), boiling stable protein (SP) genes, calcium-binding protein (CML16), 3-phosphoglycerate dehydrogenase (PGDH), UDP-glucuronic acid decarboxylase (UXS-4), amino-cyclopropane carboxylate oxidase (ACO), and hydrophobic protein (LTI6B). The role of ALDH in plant responses to pathogens is limited, but there are some recent reports of plant ALDHs involved in plant defense responses against pests and pathogens, especially during osmotic stress and drought [39]; therefore, we considered them when studying the effects of abiotic stress. The TF bHLH6 is involved in the adaptive response to various abiotic stresses [40] and in scavenging free radicals to prevent the accumulation of reactive oxygen species (ROS) [41]. This TF is involved in resistance to drought, low temperature, and salt [42], and regulates JA and ABA signaling [43]. SP is a stress-responsive protein associated with the ABA signaling pathway. It has been shown to play a key role in water stress, dehydration and heat stress [44,45]. CML16, similar to PGDH, is important for plant development and response to many stress responses, such as abiotic stress, drought, salt, low temperature, plant immunity, and oxidative stress [46,47,48,49,50,51]. We also found overexpression of UXS-4, indicating strong cell wall polysaccharide and xylan biosynthesis after the treatments [52,53], which is also involved in osmotic stress tolerance [54]. The dependence of ET metabolism in the treatments was also observed by the high expression of ACO, whose expression correlates with high ET concentration [55]. The responses to cold stress by LTI6B are closely related to changes in membrane potential [56] and accumulation of ABA. The enzyme LTI6B was up-regulated in our experiments, suggesting that treatments strengthen plants against cold and salt stress [57].

Most of the overrepresented transcripts among the top 50 were coding sequences of proteins involved in abiotic and biotic stress responses. These were JAZ proteins (TIFY9, TIFY10, TIFY11e), blue copper-binding proteins (BlueCu_1_ BS), PAL, PR2, PR6, AOS, and chemocyanin-like protein (CLP1).

The high expression of JAZ protein involved in JA and other hormone signaling pathways, including auxins, gibberellins (GAs), ABA, SA, and ET [21,22] indicates the strong hormonal stimuli of EL. Changes at the transcriptional level in the JA pathway, as the main effect of EL, were also detected by the expression of BlueCu_1_ BS, which has been described in the cotton immune response, lignin synthesis, and JA pathway [58]. The upregulation of the key enzyme AOS of the JA biosynthetic pathway supports this concept. AOS regulates the biosynthesis of JA and biologically active jasmonoyl isoleucine (JA-isole) [21], whose compounds play an important role in mediating plant responses and defense against various biotic (pathogens, insects, and herbivores) and abiotic (drought, cold, salt, heat, and heavy metal toxicity) stresses. Therefore, the enzyme AOS, as well as PAL, have received considerable attention [25]. The biosynthesis process of SA has two branches. The branch that depends on PAL involves processes outside the chloroplast, and the final product, SA, is required in response to various environmental stresses, including infection by pathogens, wounding, nutrient deficiency, UV irradiation, and extreme temperatures [23]. Therefore, this enzyme is essential for signal-triggered systemic plant resistance [24].

PR genes are key genes in the elimination of various abiotic and biotic stresses in primed plants [59,60]. We found PR6 and PR2 among the top 50 DEGs. Overexpression of these genes also proves the induced resistance during plant defense mechanism and primed status. PR6 is effective against insects and pathogens [61], and BBI-expressing plants show better performance under drought stress due to the observed lower increase in glutathione S-transferase (GST) antioxidant enzyme activity and lipid peroxidation (MDA content) [62]. PR2 is a hydrolyzing enzyme indirectly and directly involved in plant defense responses against various pathogenic fungi, bacteria, and viruses such as tomato yellow leaf virus (TYLCV) [63,64,65]. The defensive effects of EL and other compounds associated with JA have also been suggested by the overrepresented CLP1 genes, whose role has been demonstrated in wheat and which have also been associated with responses to high salinity, severe copper stress, and stripe rust [66]. The categorization of these genes according to GO revealed the differential effects of treatment combinations on defense responses. On this basis, two groups could be delineated: (i) EL, EL + SA; and (ii) EL + BABA, EL + BABA + SA. Although all treatments stimulated the metabolic processes of JA, Group (i) was found to have a stronger positive regulation of the response to herbivory through the increase in monooxigenases [67], hormones ABA, GA, ET, SA, JA, and the response to fungi and bacterial wounds after treatment with EL and EL + SA. In contrast, genes falling into these categories were downregulated in Group (ii). In this group, the response to water and desiccation stress removal were more pronounced. In addition, the response to nutrient and metal stress was associated with malate dehydrogenase [68] and iron–sulfur-cluster-binding activity [69]. Genes involved in oxidative stress response (ROS) were more expressed in Group (ii). Since ROS directly inhibits pathogen growth and can stimulate cell wall cross-linking, these processes are involved in mediating signal transduction for the expression of defense- and stress-sensitive genes [70,71].

### 3.1. Synergistic/Antagonistic Activation of JA Pathway

As described above, analysis of DEGs shows strong activation of JA-metabolic pathway genes involving the AOS gene in the top 50 DEGs, which was detected in all treated sample pairs. The key enzymes of the JA pathway are LOX, AOS, and AOC, which are localized in chloroplasts, and OPR (OPDA reductase), which is localized in peroxisomes. AOS catalyzes the dehydration of 13-hydroperoxy-octadecatrienoic acid to an unstable epoxide, which is converted to 12-oxo-phytodienoic acid (OPDA) by AOC. Due to the acute instability of the epoxide, AOS and AOC are likely functionally and physically linked [21]. Transcription of AOS occurs after biotic stress, such as wounding, and the promoter can be activated by a variety of signals, including jasmonic acid, wounding, OPDA, and SA. The regulation of AOS gene expression is mainly controlled by JA signaling [72]. Since JAZ proteins involving TIFY9 are associated with hormonal signaling pathways, especially JA and other hormones such as auxins, GAs, ABA, SA or ET [21,22], it is logical that we found TIFY genes among the top 50 DEGs induced after the treatments studied. Analyzing the RPM levels of key enzymes of JA pathway genes, we found that SA, in addition to EL-induced LOX-, AOS gene expression, and AOC, were induced when BABA or SA was added to the EL-, BABA + SA combination treatment, showing antagonism (Figure 4). OPR gene expression was very low in the samples tested and could not be evaluated. TIFY9 expression was higher after treatment with EL, and additional treatments decreased the effect. This may be due to the fact that EL, as a mixture of 11 plant extracts, contains numerous plant hormones that stimulate the promoter of JAZ proteins (Figure 5). The effect of EL on TIFY9 suggests that neither SA nor JA (triggered by BABA) have a higher inductive effect on TIFY motif proteins, but EL may contain other hormonal components that have a stronger JAZ-inducing transcription. Since the enzymes with increased gene expression are bound to the chloroplast structure, the decreased expression of photosynthetic enzyme genes in the treated samples could be explained by the plant cells using energy to strengthen the JA pathway rather than to increase photosynthetic activity.

### 3.2. Synergistic/Antagonistic Activation of SA Pathway

The RPM indices of SA pathway genes were compared with samples from all treatments after 2 days. The key enzymes of the SA pathway are isochorismate synthase (ICS) and PAL, which catalyze the two synthetic pathways of SA separately. ICS, which is localized in the chloroplast, catalyzes the isomerization of chorismate to isochorismate, an essential precursor of the biosynthesis of the electron transmitter phylloquinone of photosystem I. These are the ICS-dependent pathways for the biosynthesis of SA. The other pathway of SA is initiated by PAL, which deaminates phenylalanine to trans-cinnamic acid, leading to the conversion of benzoic acid as a precursor for SA in the PAL-dependent SA pathway [73]. Complementary treatments with EL stimulate the PAL-dependent pathway, but it was also observed that the combined treatment worsens the effect of EL. The positive effect of BABA on the induction of ICS and PAL genes is evident; however, we found suppression of these genes after the addition of exogenous SA (Figure 6). Moreover, the SA-inducing effect of the combined application of BABA and SA is striking compared to EL, which has exactly the opposite effect to the JA pathway.

A summary of the synergistic and antagonistic effects of the studied combinations on the key genes of the SA and JA pathways is shown in Table 2. According to this summary of the data from the DEG and RPM analyses, the synergistic effect of the SA pathway was identified in all combinations. However, the antagonistic effect on the genes of the JA pathway was found in the case of EL + BABA and EL + BABA + SA, which is due to the fact that BABA and SA do not stimulate these genes per se.

## 4. Materials and Methods

### 4.1. Treatment of Barley Seedlings by Priming Active Agents

**Figure 7:**
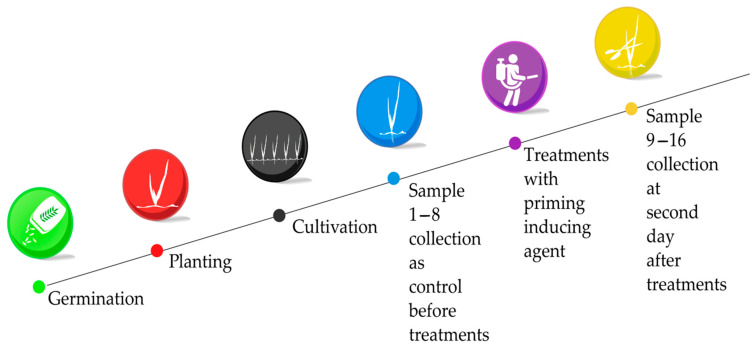
Experimental design of phytotron experiments of cultivation and sample collection.

The synergistic effects of the exogenous treatments of the combinations SA, BABA, and EL were examined (Table 3) by sampling at two time points (Figure 7), resulting in 16 treatment samples. Three biological replicates of each combination were collected and used to prepare Illumina Gex libraries for RNA-Seq, using the NextSeq550 sequencing platform. Transcriptional profile analysis for the whole genome and comparison of DEGs were performed by calculating the mathematical distance metrics of the matrix of transcript abundances. The identity of the most highly expressed samples and the differentially expressed genes was then assessed.

The plants were cultivated in a controlled environment using an MLR-352H Panasonic growth chamber. The temperature conditions were as follows: on the first day and night, the temperature was maintained at 25 °C. From the second day to the sixteenth day, the daytime temperature was set to 25 °C, while the nighttime temperature was lowered to 15 °C. The plants followed a photoperiod of 10 h of light, followed by 14 h of darkness, and the relative humidity was maintained at a constant level of 60 ± 5%.

The priming inducers used for treatment were Na-SA and BABA, prepared in a solution with a concentration of 300 μM and 25 mM, respectively, which was added to a solution of EL prepared at a ratio of 0.1 mL per 100 mL of water. The treatment application was carried out using a Bürkle pressure sprayer equipped with an adjustable spray jet, with a nozzle diameter of 0.8 mm. The Arabidopsis leaves were sprayed from multiple angles until they were visibly wet, ensuring complete coverage.

### 4.2. RNA Isolation and Sequencing

Approximately 30 mg of plant tissue was added to a 1.5-mL Eppendorf LoBind tube containing 1.7–2.1 mm diameter glass beads (Carl Roth, Karlsruhe, Germany) and 100 μL of TRI reagent (Zymo Research, Irvine, CA, USA). The Eppendorf tube was firmly connected to a SILAMAT S5 vibrator (Ivoclar Vivadent, Schaan, Liechtenstein) to crush and homogenize the tissue for 2 × 15 s. The tissue was then placed in the tube. Total RNA was extracted using the Direct-zol™ RNA MiniPrep System (Zymo Research, Irvine, CA, USA), according to the manufacturer’s protocol. RNA integrity numbers and RNA concentration were determined using the RNA ScreenTape System with 2200 Tapestation (Agilent Technologies, Santa Clara, CA, USA) and RNA HS Assay Kit with Qubit 3.0 Fluorometer (Thermo Fisher Scientific, Waltham, MA, USA), respectively. Illumina NextSeq550 libraries were prepared according to the manufacturer’s instructions, and samples were multiplex-sequenced in the same sequencing run using dual-indexing adapters. For library amplification, adapter-selective PCR was performed. Before sequencing, the fragment size distribution and purity of the samples were checked using the Agilent 2100 Bioanalyzer (Agilent Technologies, Santa Clara, CA, USA). Libraries were sequenced using a single-end option, and the final output consisted of 14–26 M x 85 base pairs’ (bp) long reads (1.19–2.21 Gbp). Raw sequences were stored in the National Center for Biotechnology Information (NCBI) database under bioproject PRJNA721578 (https://www.ncbi.nlm.nih.gov/bioproject/PRJNA721578; SRA Accession Numbers: SRX10600133-SRX10600148), accessed on 1 September 2022.

### 4.3. Data Preprocessing, De Novo Assembly, Gene-Level Quantification, and DEG Determination

Sequence data preprocessing, gene-level quantification, and DEG analysis were performed as described in our previous publications [20,74]. The tools used in bioinformatic processing are summarized in Table 4.

After quality control and filtering out low-quality reads and base pairs, a de novo reference transcript dataset was reconstructed with Trinity v2.15.1 [75] using the combined read-set of 16 samples. The resulting transcriptome was deposited at Mendeley Data (https://data.mendeley.com/datasets/68zy55gt62/1, accessed on 31 March 2023). CountTable was based on RSEM, a software package that quantifies transcriptome expression to estimate transcript abundance. The matching of reads to reference transcript sequences and calculation of relative abundances was performed using the Bowtie2 aligner, with parameters explicitly chosen for RNA-Seq quantification. Because RNA-Seq reads cannot always be uniquely assigned to a single gene or isoform, the assignment of multi-mapping reads to transcripts was performed using an expectation maximization approach [76,77]. Pairwise differential expression analysis between experimental conditions (1 vs. 10–16) was performed using NOISeq [78]. This tool uses a nonparametric approach to identify differentially expressed genes from RNA-Seq count data. It creates a null or noise distribution of count changes by contrasting fold-change differences (M) and absolute expression differences (D) for all genes in samples within the same condition. This reference distribution is then used to assess whether the M and D values calculated between two conditions for a given gene are likely part of the noise or represent true differential expression. Data were visualized in a heat map after extracting the top 50 DEGs in the sample pairs studied. DEGs were ranked according to the false discovery rate (FDR) calculated by NOIseq. Dendrograms were reconstructed by a hierarchical clustering method using the Euclidean distance calculated between genes as input.

### 4.4. Functional Annotation of Transcripts

Functional annotation of novel sequences was performed using EggNOG-mapper [79], which uses precomputed eggNOG-based orthology mappings to make predictions for functional annotation more accurate than traditional homology searches by avoiding the transfer of annotations from paralogs. Functional annotation results are available in the Mendeley Data Repository (https://data.mendeley.com/datasets/68zy55gt62/1, accessed on 31 March 2023).

### 4.5. Enrichment Analysis (Fisher’s Exact Test)

For the statistical evaluation of annotation differences between 2 sets of sequences, Fisher’s exact test was performed using the FatiGO package integrated in Blast2GO [80]. The functional annotation of the enriched gene IDs was performed based on AnnotationTable, and using in-house developed software.

### 4.6. Calculation of RPM Index

The transcript sequences of selected genes were extracted from the reference transcriptome, and the quality-filtered reads of the 16 samples were realigned to the coding sequences. Read alignment was performed using Bowtie2. Individual reads per million (RPM)-mapped reads, also known as CPM, were calculated for each gene (Equation (1)).

(1) $$RPM=\frac{(mapped\;reads\;pera\;gene)\cdot 10^{6}}{total\;mapped\;reads}$$

In some specific RNA-seq protocols, particularly apartment RNA-seq methods, reads are generated from only one end of the RNA molecule, regardless of length. The RPM gene expression index does not take into account the length of the transcript. After normalization, it is a suitable gene expression unit for sequencing protocols that generate reads regardless of gene length [81]. We used the values of RPM to determine the individual expression levels of the enzyme sequences active in each treatment at a given time point, i.e., their actual expression levels. The values of RPM reflect the individual expression levels of the enzyme sequences triggered by the treatments applied at a given time point [82].

## 5. Conclusions

In the present study, a green-technology-based plant conditioning agent was supplemented with a small amount of substances that produced a priming effect, which is a novelty in the field of plant protection. Gene induction analysis initially showed a potential *trans*-priming effect of the tested compounds in combinations, indicating the possibility of a more effective strategy for organic plant protection.

Accordingly, the plant conditioning effect of EL may be enhanced by the addition of other hormones or priming-active compounds; however, the combined addition of BABA and SA may result in antagonism. Because EL itself triggers the JA/ET and SA responses, additional compounds may be added to EL depending on the type of stress being amplified—disease-related stress or environmental stress. Treatments of (monocotyledonous) barley showed that the addition of BABA helped alleviate salt, water, osmotic, or metal stress. The addition of SA may increase stress responses to fungal, bacterial, or insect attacks, and both may contribute to the *trans*-priming state of plants.

## Figures and Tables

**Table 1:** Genes of the top 50 DEGs involved in phytohormonal stress responses mediated by the signaling pathways SA and BABA. Sequences annotated as JAZ proteins with TIFY motif, PAL, and AOS genes were selected for further analysis.

*C*	*EL*	*EL + BABA*	*EL + SA*	*EL + SA + BABA*	Gene name	Function
*Down*	*UP*	*Down*	*UP*	*UP*	*TIFY9* *TIFY10* *TIF11e*	Are involved in JA and other hormone signaling pathways, including auxins, gibberellins (GAs), ABA, SA, and ethylene (ET) [21,22]
*Down*	*UP*	*UP*	*UP*	*UP*	*PAL*	PAL gene expression responds to a variety of environmental stresses, including pathogen infection, wounding, nutrient depletion, UV irradiation, and extreme temperatures [23]. It is involved in the biosynthesis of SA, essential signal involved in plant systemic resistance [24].
*Down*	*UP*	*UP*	*UP*	*UP*	*AOS*	It has a key role in the synthesis of JA and biologically active jasmonoyl-isoleucine (JA-Ile) [21]. It plays important roles in the mediation of plant responses and defenses to various biotic (pathogen, insect, and herbivore) and abiotic (drought, cold, salt, heat, and heavy metal toxicity) stresses therefore have received extensive research attention [25].

**Table 2:** Summary of the change in regulation of JA and SA metabolic genes as a result of the analyses of DEG and RPM (analyses are indicated after the gene name).

		SA-pathway			JA-pathway					
		PAL DEG	PAL RPM	ICS RPM	TIFY DEG	TIFY RPM	AOS DEG	AOS RPM	LOX RPM	AOC RPM
Control	1	DOWN	UP	DOWN	DOWN	DOWN	DOWN	DOWN	DOWN	DOWN
SA	10	UP	DOWN	DOWN	DOWN	DOWN	DOWN	DOWN	UP	DOWN
BABA	11	UP	UP	UP	DOWN	DOWN	DOWN	UP	UP	DOWN
BABA + SA	12	UP	UP	UP	UP	UP	UP	DOWN	DOWN	DOWN
EL	13	UP	UP	UP	UP	UP	UP	UP	UP	UP
EL + SA	14	UP	UP	UP	UP	UP	UP	UP	UP	UP
EL + BABA	15	UP	UP	UP	DOWN	UP	UP	UP	UP	DOWN
EL + BABA + SA	16	UP	UP	UP	UP	UP	UP	DOWN	UP	DOWN

**Table 3:** Number and identification of samples of barley seedlings treated with priming agents and their combinations. Samples were taken on Day 0 (15 min after treatment) and two days after treatments.

Treatment	Day 0	Day 2
Control	1 HV_1)	9 (HV_9)
SA	2 (HV_2)	10 (HV_10)
BABA	3 (HV_3)	11 (HV_11)
BABA + SA	4 (HV_4)	12 (HV_12)
EL	5 (HV_5)	13 (HV_13)
EL + SA	6 (HV_6)	14 (HV_14)
EL + BABA	7 (HV_7)	15 (HV_15)
EL + BABA + SA	8 (HV_8)	16 (HV_16)

**Table 4:** Applied bioinformatics tools used during RNA-Seq data processing.

Process	Software	Web Page
Quality control	FastQc V0.11.9	https://github.com/s-andrews/FastQC (8 June 2023)
Filtering	Trimmomatic v0.39	https://github.com/usadellab/Trimmomatic/releases (8 June 2023)
De novo assembly	Trinity v2.15.1	https://github.com/trinityrnaseq/trinityrnaseq/wiki (8 June 2023)
Functional annotation	EggNOG-Mapper V5	http://eggnog-mapper.embl.de/ (8 June 2023)
RNA-Seq alignment	Bowtie2 v2.4.5	https://bowtie-bio.sourceforge.net/bowtie2/index.shtml (8 June 2023)
Expression quantification	RSEM v1.3.3	https://deweylab.github.io/RSEM/ (8 June 2023)
Distribution of GO term	Blast2GO v6.0	https://www.blast2go.com/ (8 June 2023)

## Data Availability

The raw reads were deposited in the National Center for Biotechnology Information (NCBI) database under the BioProject PRJNA721578 (https://www.ncbi.nlm.nih.gov/bioproject/PRJNA721578) accessed on 1 September 2022. RNA sequencing of phytotron experiments, SRX10600133-SRX10600148. De novo reference transcripts, Annotation Table and CountTable are deposited in Mendeley Data and available at: https://data.mendeley.com/datasets/68zy55gt62/1, accessed on 8 June 2023).

## Supplementary Materials

The following supporting information can be downloaded at: https://www.mdpi.com/article/10.3390/plants12122308/s1, Figure S1: Heatmaps; Table S1: Functional description of Top50DEGs.
