# Supplementary material for: Supplementation of the Plant Conditioner ELICE Vakcina® Product with β-Aminobutyric Acid and Salicylic Acid May Lead to Trans-Priming Signaling in Barley (Hordeum vulgare)
Source: Plants (Basel). 2023 Jun 14;12(12):2308. doi: 10.3390/plants12122308 (PMC10305027; doi:10.3390/plants12122308)
Supplement: Supplementary file 1 [file plants-12-02308-s001.zip › supplementary/TableS1.pdf]

TableS1
Functional description of Top50DEGs

Gene product	*Abbreviation*	*Function*
**Photosynthesis**		
Far-red impaired response1	FAR1	Required for chlorophyll biosynthesis (Ma and Li, 2018)<br>Enhances the expression of defense-responsive genes (Tang et al., 2012)<br>Binds to the ABI5 promoter and activate its transcription thus mediating ABA signal transduction and abiotic stress responses (Skubacz et al., 2016)
NifU-like protein 2, chloroplastic	NIFU	Required for the assembly of photosystem I complex (Leon et al., 2003)
**Abiotic stress and biotic stress**		
JAZ proteins*	TIFY9<br>TIFY10<br>TIFY11e	Are involved in JA and other hormone signaling pathways, including auxins, gibberellins (GAs), ABA, SA, and ethylene (ET) (Turner et al, 2002, Solano et al, 2003)
Far-red impaired response1	FAR1	Required for hlorophyll biosynthesis (Ma and Li, 2018)<br>Enhances the expression of defense-responsive genes (Tang et al., 2012)<br>Binds to the ABI5 promoter and activate its transcription thus mediating ABA signal transduction and abiotic stress responses (Wang et al., 2016)
Blue-copper binding proteins	BlueCu_1_BS	Regulate cotton immune response through lignin synthesis and JA signaling pathway (Zhu et al, 2018)
Phenylalanine ammonia lyase*	PAL	PAL gene expression responds to a variety of environmental stresses, including pathogen infection, wounding, nutrient depletion, UV irradiation, and extreme temperatures (Huang et al., 2010)<br>It is involved in the biosynthesis of SA, essential signal involved in plant systemic resistance (Kim and Whang, 2014)
Bowmann-Birk type-proteinase inhibitor	BBI<br>PR6	Play a role in plant defense against insects and pathogens (Xie et al., 2021)<br>*BBI*-expressing plants exhibit better performance under drought stress due to the observed reduced increase in antioxidant enzymes activity (GST) and reduced MDA content. (Malefo et al., 2020)
Endo-1,3(4)-beta-glucanase 2	PR2	A hydrolyzing enzymes that indirectly and directly participate in the plant defense response against various pathogenic fungi, bacteria and viruses like tomato yellow leaf curl virus (TYLCV) (Mestre et al. 2017; Wang et al.2020; Hata et al. 2021).
Allene-oxyde sinthase*	**AOS**	It has a key role in the synthesis of JA and biologically active jasmonoyl-isoleucine (JA-Ile) (Turner et al., 2022).<br>It play important roles in the mediation of plant responses and defenses to various biotic (pathogen, insect, and herbivore) and abiotic (drought, cold, salt, heat, and heavy metal toxicity) stresses therfore have received extensive research attention (Wang et al., 2021)

Chemocyanin-like protein	CLP1	It plays positive roles in wheat response to high-salinity, heavy cupric stress and stripe rust (Feng et al., 2013)
**Biotic stress**		
Papain-like cistein proteases	PLCPs	Are required for full resistance of plants to various pathogens (Linde et al, 2012)<br>Are targeted by secreted pathogen effectors to suppress immune responses (Ilyas et al, 2015)<br>Are subject to a co-evolutionary host–pathogen arms race (Kashani and Horn 2010).<br>Induce a broad spectrum of defense responses including plant cell death.<br>(Willamil et al., 2016)
Thionins	BTH7	Are plant-specific antimicrobial peptides isolated from numerous plant species (Plattner et al., 2015)
Catalase2	Cat-2	Serves to protect cells from the toxic effects of hydrogen peroxide (Song et al., 2021)<br>It is involvedin response to biotic stimulus (Zhang et al., 2021)<br>CAT2 expression also plays an important role in plant immunity(Baker et al., 2023).<br>It promotes JA biosynthesis by facilitating direct interaction of the JA biosynthetic enzymes ACX2 and ACX3, and thus SA repression of CAT2 inhibits JA accumulation (Yuan et al., 2017)
**Abiotic stress**		
Aldehyde dehidrogenase family protein	ALDH	plant responses to pathogens are limited, but a few recent report on plant ALDHs involved in the plant defense response against pests, pathogens, but mainly in osmotic stress, drought stress (Tola et al., 2021)
TF bHLH family	bHLH6	Involved in adatptive response to variuos abiotic stress (Qua net al., 2021)<br>Involved in free radical scavange and elimination of ROS accumulation (Ji et al., 2016)<br>Participate in drought resistance, law temperature and salt resistance (Lu et al., 2022)<br>Involved in JA and ABA signaling (Aleman et al., 2016)
Boiling stable protein	SP	It is a stress responsive protein associated with ABA pathway and water, desiccation, heatstress (Sharma et al., 2019, Rakhra et a., 2017)
Calcium-binding protein	CML16	It is involved to plant development and response to stresses such as abiotic, drought stress, plant immunity, oxidative stress (Zeng et al., 2015, Ranty, 2016., Zhu et al., 2017, Magnan et al., 2018, Delk et al, 2005)
3-phosphoglycerate dehidrogenase	PGDH	Presumably it is involved in abiotic stress, such as salt, drought, oxidative and low temperature,in higher plants, however its role is largely unexplored (Wang et al., 2021)
UDP-glucuronic acid decarboxylase	UXS-4	It is involved in cell wall polysaccharides (Xu et al. 2023), xylan biosynthesis (Ruan et al, 2022) and osmotic stress tolerance (Shen et al., 2022)
Amino-cyclopropane-carboxylate oxidase	ACO	It is involved in abiotic stress and its expression is associated to ETH concentration (Napieraj et al., 2023)
Hydrophobic protein LTI6B	LTI6B	Plays a role in the regulation of membrane potential (Morsy et al., 2005)

		Involved in response to ABA and response to cold and salt stress (Razzaque et al., 2017)
