# Supplementary figures and images for: Supplementation of the Plant Conditioner ELICE Vakcina® Product with β-Aminobutyric Acid and Salicylic Acid May Lead to Trans-Priming Signaling in Barley (Hordeum vulgare)
Source: Plants (Basel). 2023 Jun 14;12(12):2308. doi: 10.3390/plants12122308 (PMC10305027; doi:10.3390/plants12122308)

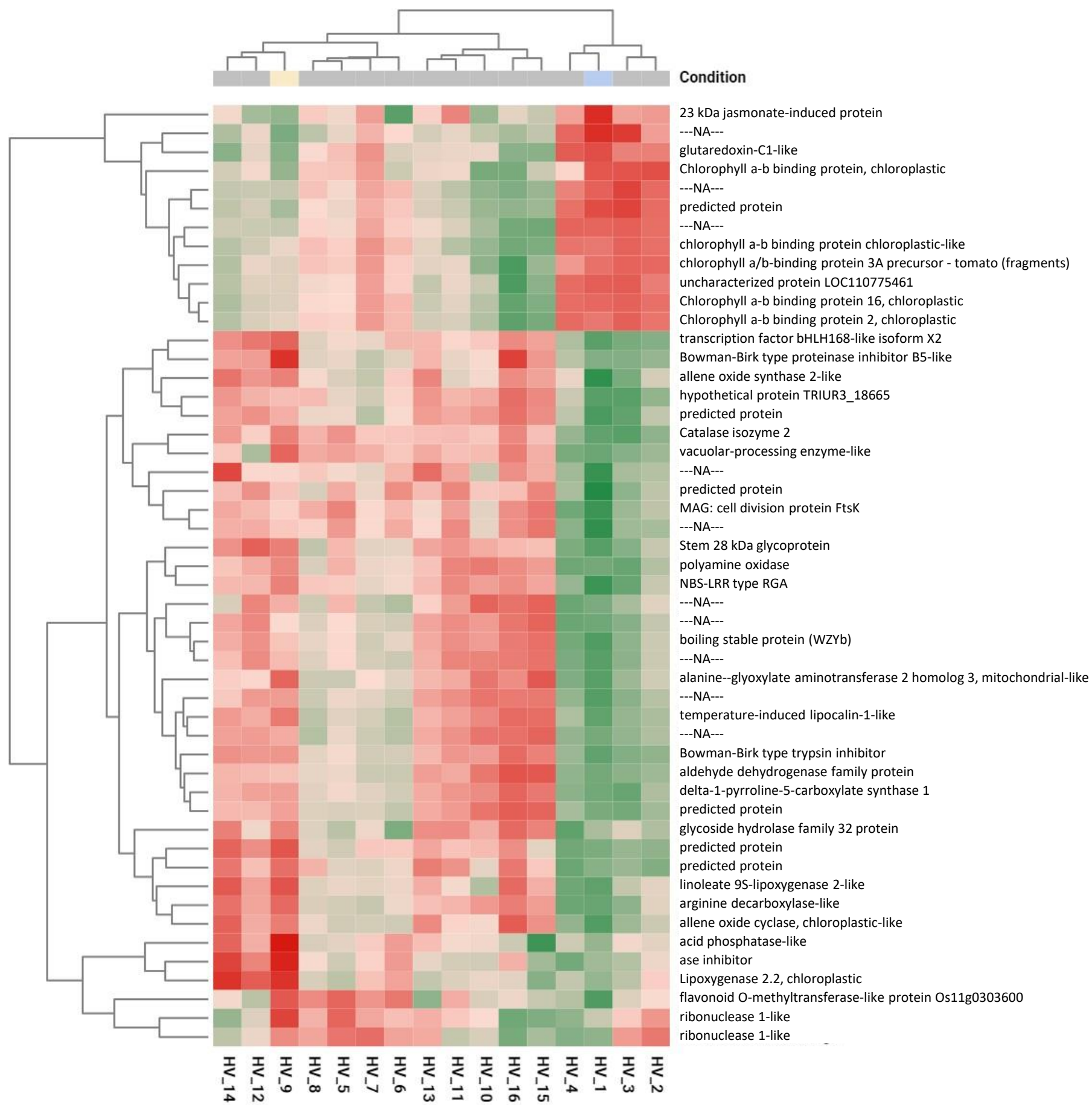

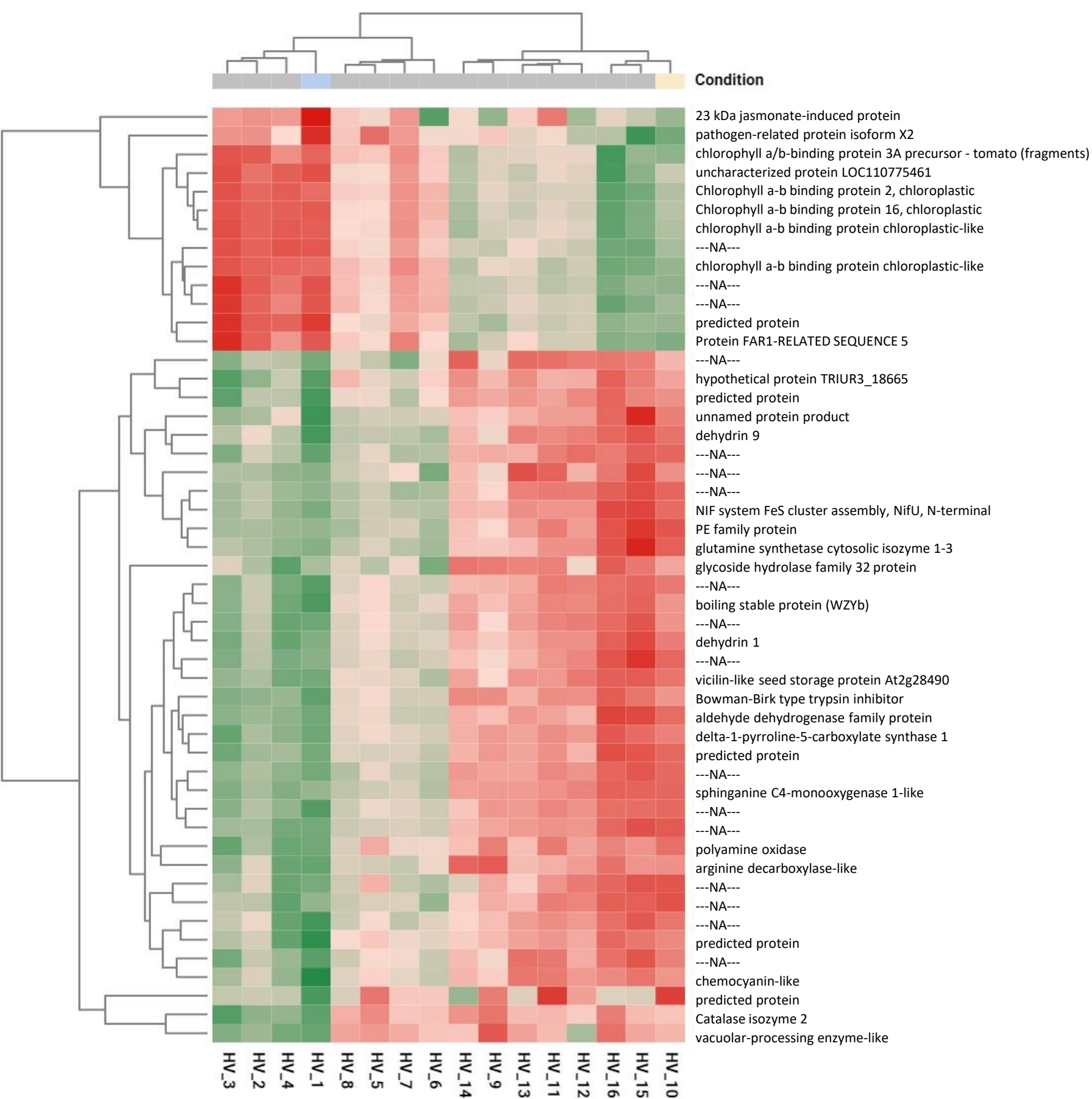

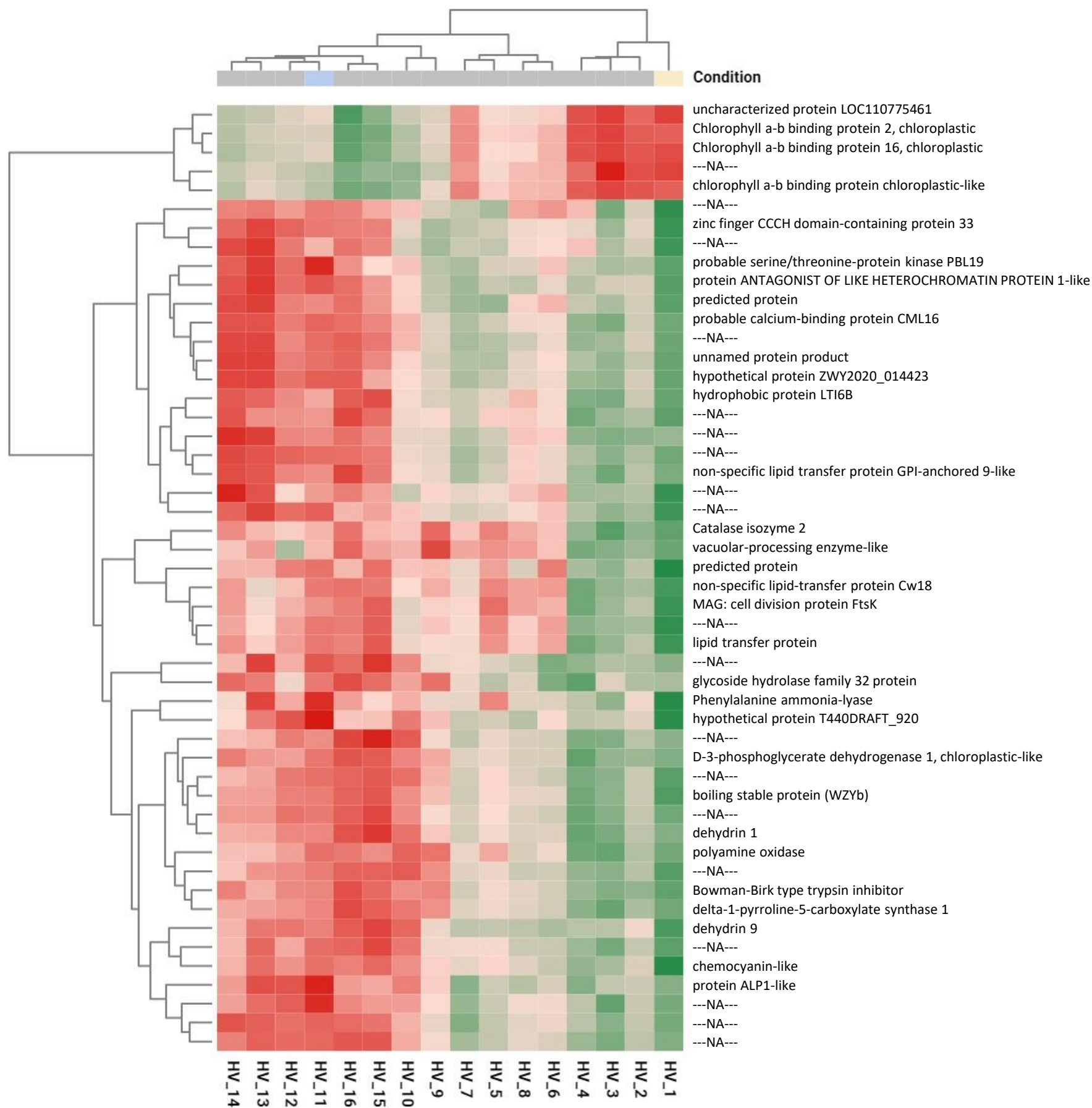

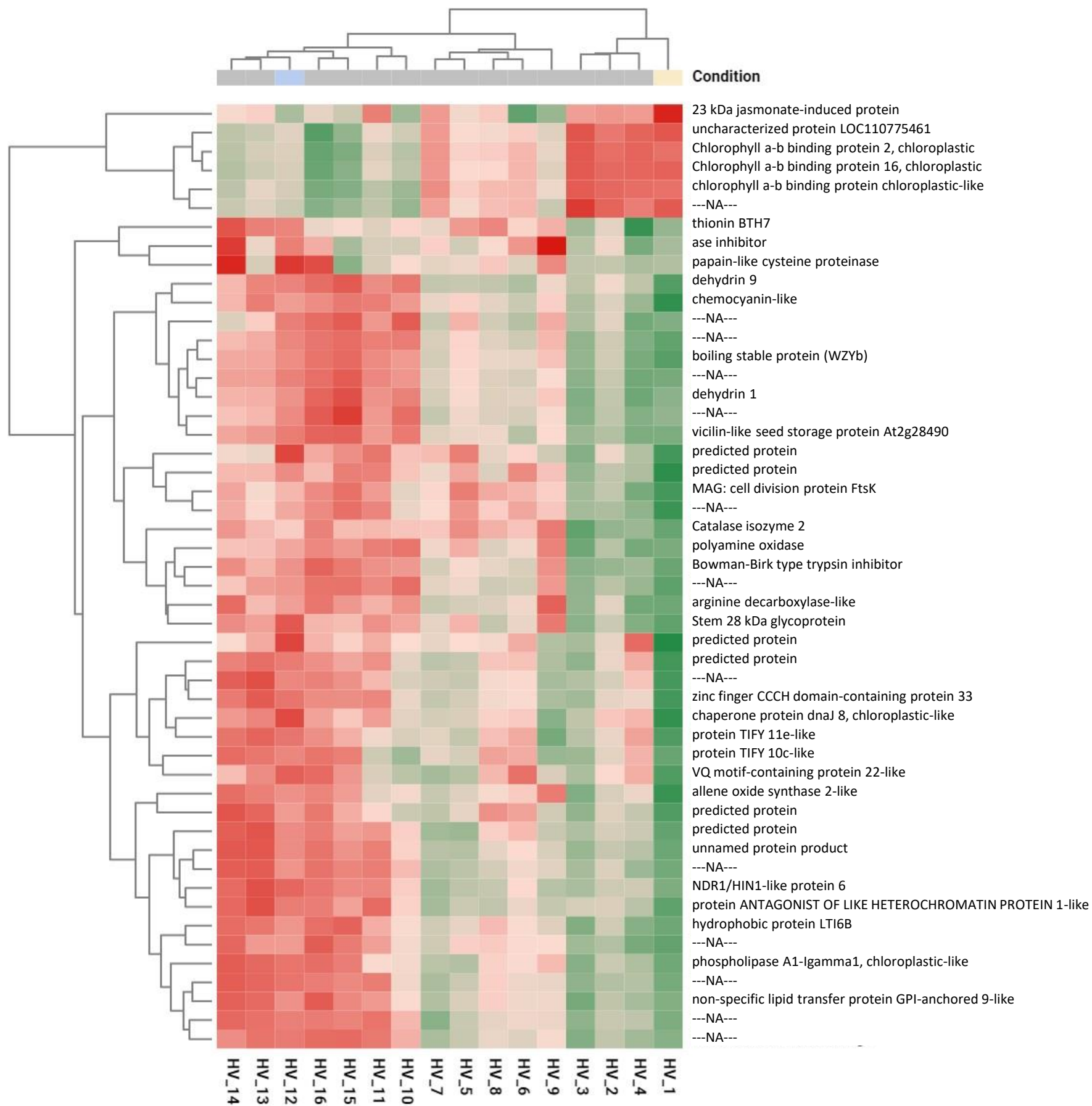

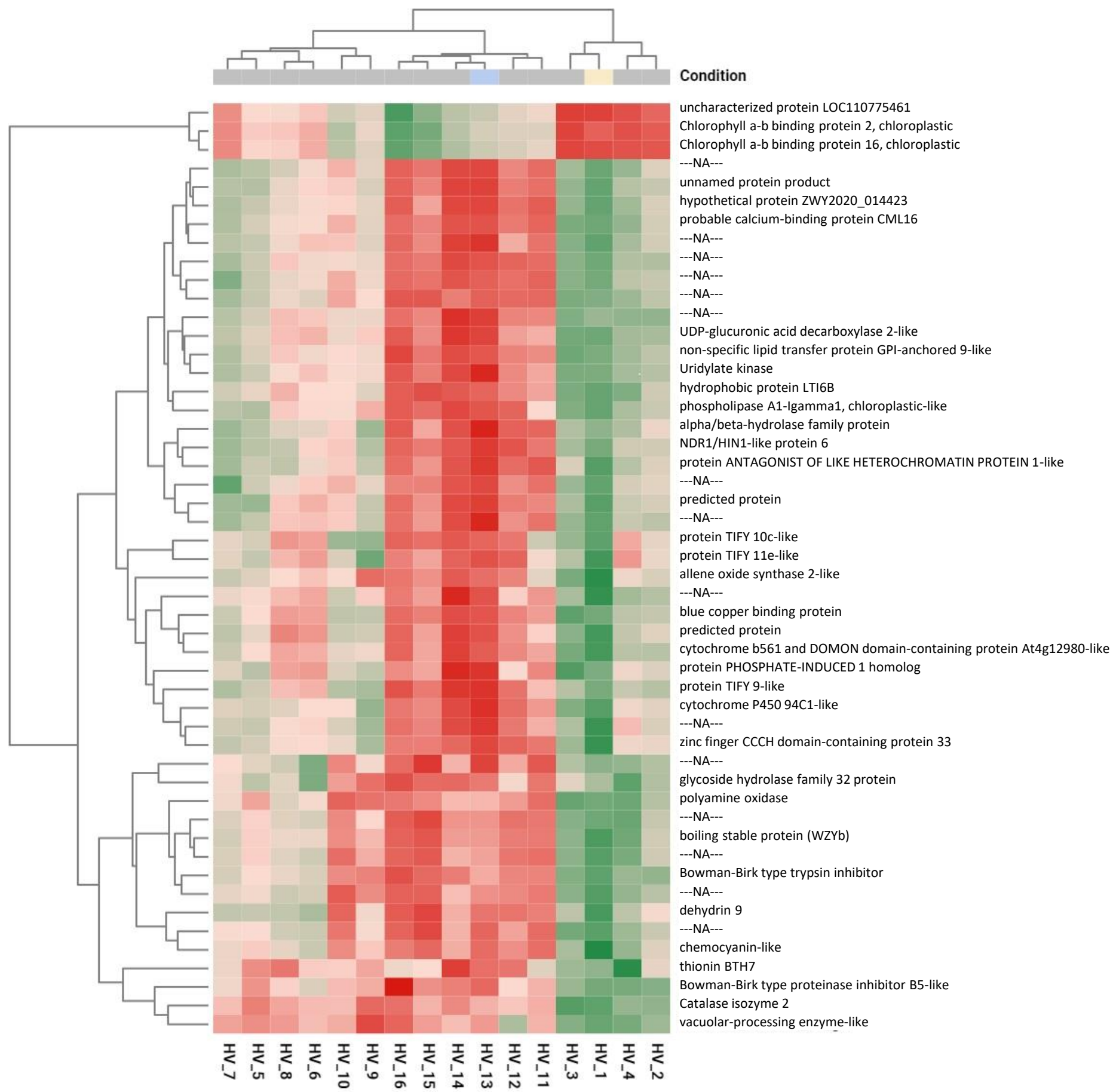

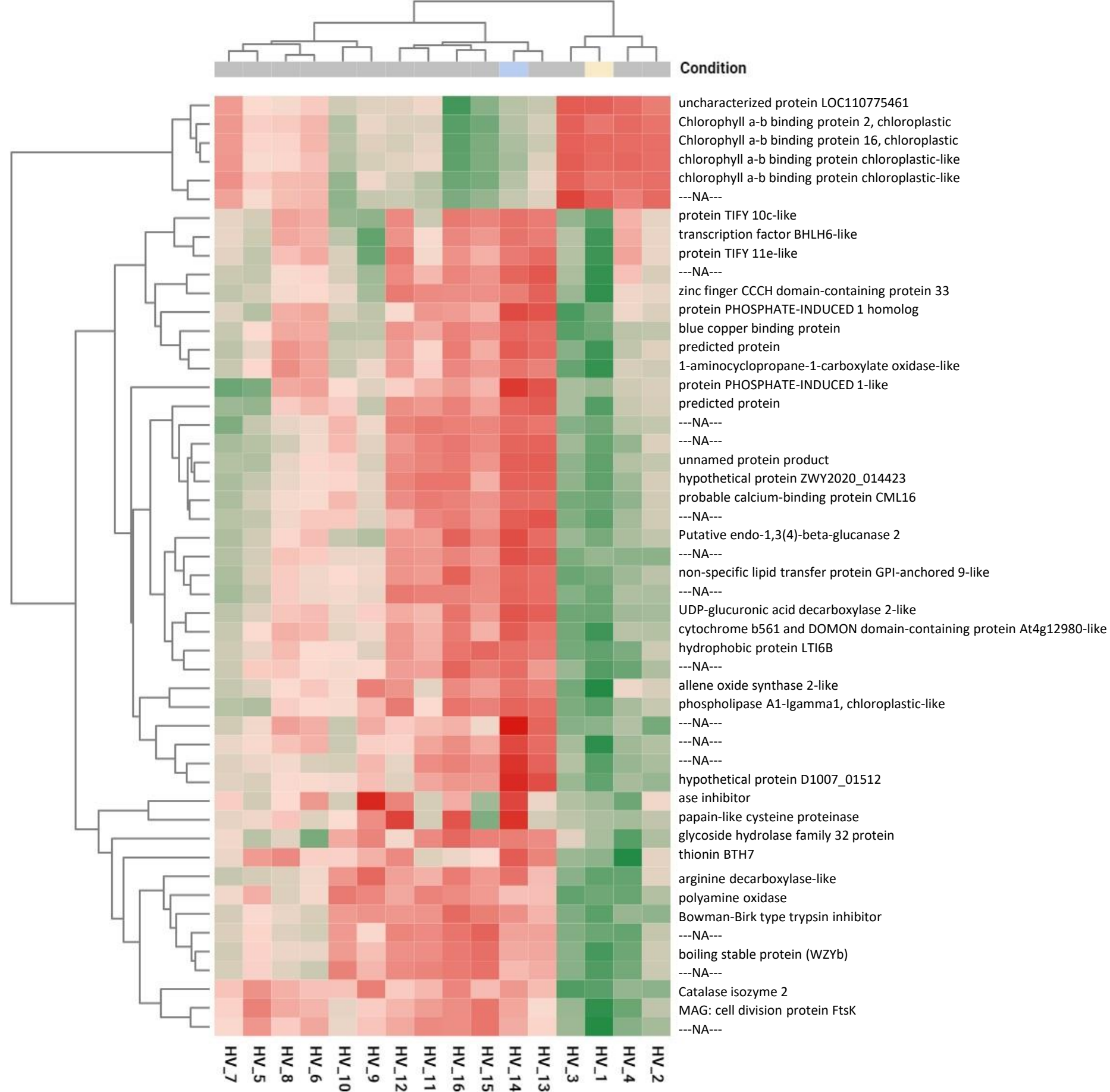

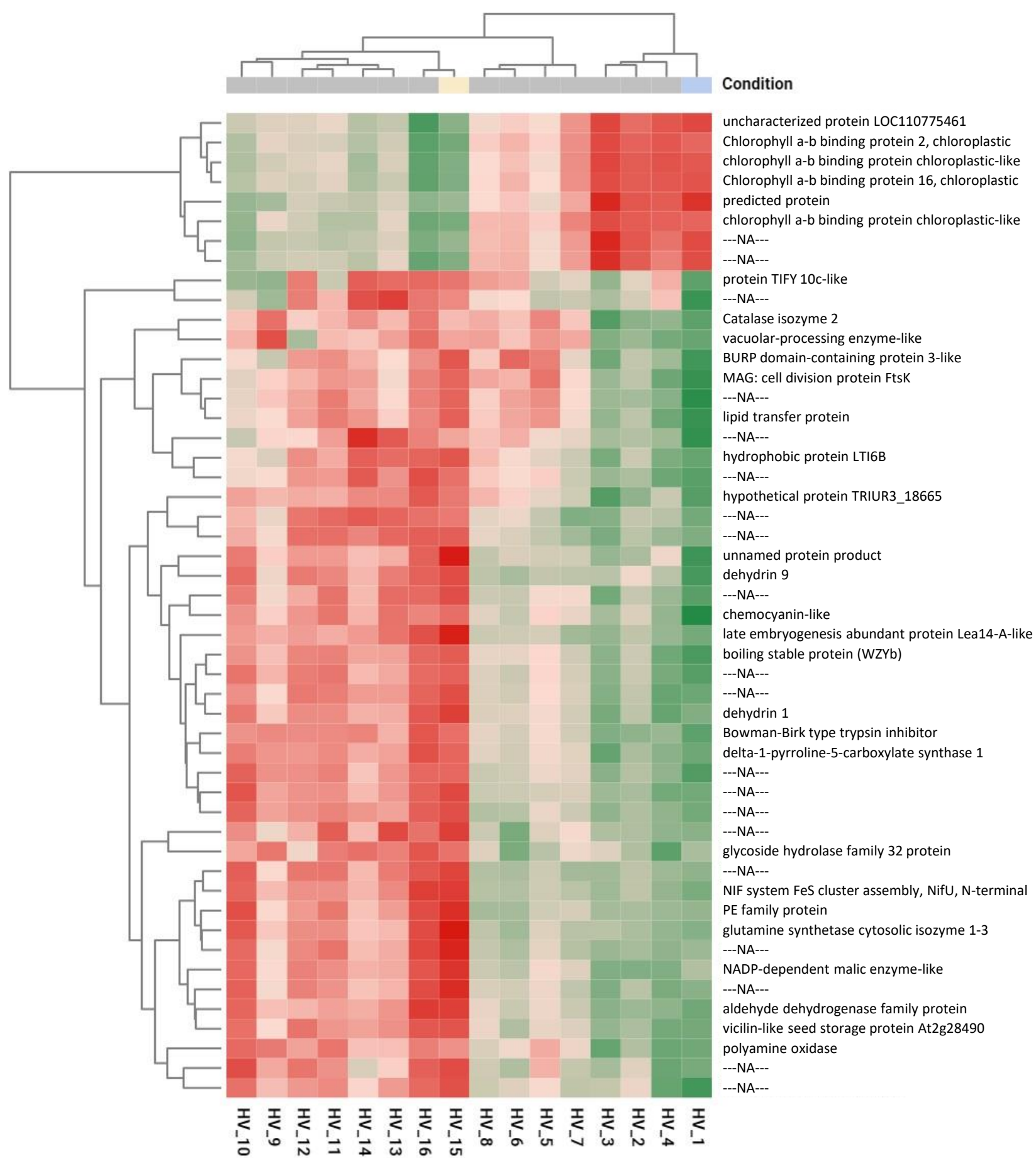

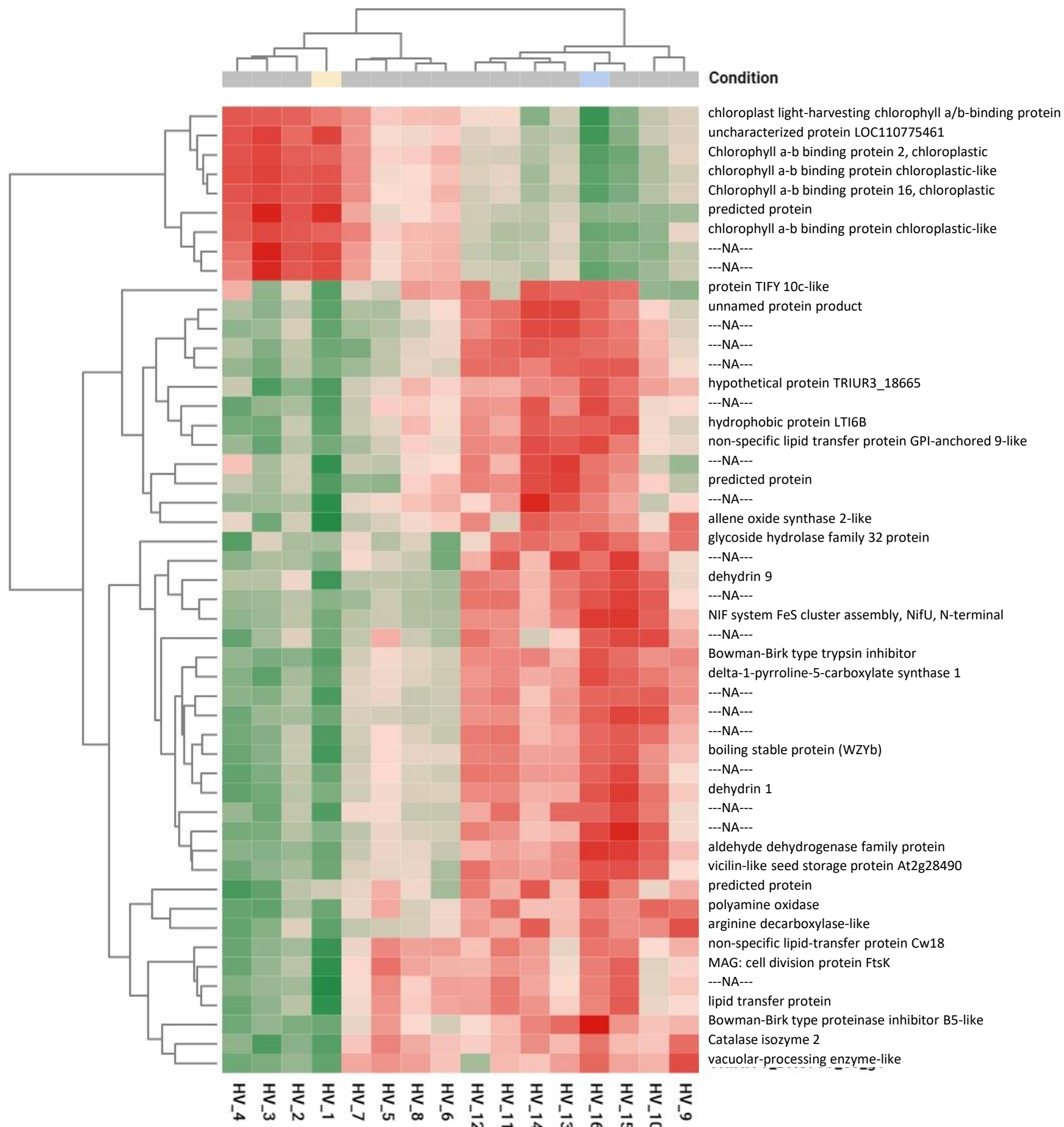

Supplement: Supplementary file 1 [file plants-12-02308-s001.zip › supplementary/FigureS1.pdf]
